# RNA-seq analysis of PHD and VHL inhibitors reveals differences and similarities to the hypoxia response.

**DOI:** 10.12688/wellcomeopenres.15044.1

**Published:** 2019-01-29

**Authors:** Julianty Frost, Alessio Ciulli, Sonia Rocha

**Affiliations:** 1 Biochemistry-Institute of Integrative Biology, University of Liverpool, Liverpool, L697ZB, UK; 2 Division of Biological Chemistry and Drug Discovery, School of Life Sciences, University of Dundee, Dundee, DD15EH, UK

**Keywords:** Hypoxia, HIF, PHDs, VHL, FG-4592, IOX2, VH298, RNA-seq.

## Abstract

**Background:** Hypoxia-inducible factor (HIF) transcription factors are well known to control the transcriptional response to hypoxia. Given the importance of cellular response to hypoxia, a number of pharmacological agents to interfere with this pathway have been developed and entered pre-clinical or clinical trial phases. However, how similar or divergent the transcriptional response elicited by different points of interference in cells is currently unknown.

**Methods: **We performed RNA-sequencing to analyse the similarities and differences of transcriptional response in HeLa cells treated with hypoxia or chemical agents that stabilise HIF by inhibiting components of the hypoxia signalling pathway – prolyl hydroxylase (PHD) inhibitor or von Hippel–Lindau (VHL) inhibitor.

**Results: **This analysis revealed that hypoxia produces the highest changes in gene transcription, with activation and repression of genes being in large numbers. Treatment with the PHD inhibitor IOX2 or the VHL inhibitor VH032 led mostly to gene activation, majorly via a HIF-dependent manner. These results were also confirmed by qRT-PCR using more specific and/or efficient inhibitors, FG-4592 (PHDs) and VH298 (VHL).

**Conclusion: **PHD inhibition and VHL inhibition mimic gene activation promoted by hypoxia via a HIF-dependent manner. However, gene repression is mostly associated with the hypoxia response and not common to the response elicited by inhibitors of the pathway.

## Introduction

Hypoxia, or reduced oxygen availability, is associated with many physiological processes, such as embryonic development and high altitude living but also pathological processes such as stroke and cancer ( Rocha, 2007). A major regulator of oxygen sensing and response is the family of transcription factors called hypoxia-inducible factors (HIFs). HIFs are activated in response to hypoxia to initiate a transcriptional program, and ultimately restore oxygen homeostasis and promote cell survival ( Kenneth & Rocha, 2008). HIFs are heterodimeric transcription factors composed of a constitutively stable β-subunit (HIF-1β) and an oxygen-labile α-subunit (HIF-α) ( Kenneth & Rocha, 2008). HIF-α is encoded by three different genes: *HIF-1α*, *HIF-2α* and *HIF-3α*, and each could function differently depending on tissue localisation ( Kenneth & Rocha, 2008). HIF-α is rapidly degraded by the proteasome under normal oxygen levels as prolyl hydroxylase domain (PHD) enzymes and factor inhibiting HIF (FIH) utilise molecular oxygen as a co-factor, in addition to Fe ^2+^ and 2-oxoglutarate, to hydroxylate HIF-α proteins ( Kenneth & Rocha, 2008). Hydroxylated prolines on HIF-α create a recognition site with a substantial increase in affinity over the parent protein containing unmodified proline, for the E3 ubiquitin ligase, von Hippel–Lindau (VHL) tumour suppressor that poly-ubiquitinates HIF-α, targeting the protein for proteasomal degradation ( Hon *et al*., 2002). In hypoxia, however, HIF-α evades degradation and is stabilised as a result of insufficient oxygen molecules for PHDs to function. The accumulated HIF-α dimerises with HIF-1β and binds to the consensus motif hypoxia response elements (HREs) of HIF target genes to activate the expression of a wide range of genes associated with key biological processes including metabolism, angiogenesis, cell differentiation, apoptosis and autophagy, for adaptation to hypoxia ( Liu *et al*., 2012).

In addition to the physiological inducer of low oxygen, HIF can be activated by chemical agents that mimic or inhibit components of the hypoxia signalling pathway, including Fe ^2+^ substitutes ( Wang & Semenza, 1993; Xi *et al*., 2004), Fe ^2+^ chelators ( Eltzschig *et al*., 2014), 2-oxoglutarate mimics ( Chan *et al*., 2016; McDonough *et al*., 2005), inhibitors of PHDs ( Chan *et al*., 2015; Chowdhury *et al*., 2013; Locatelli *et al*., 2017), and more recently, inhibitors of VHL ( Buckley *et al*., 2012; Frost *et al*., 2016; Galdeano *et al*., 2014; Soares *et al*., 2018). Pharmacological stabilisation of HIF could provide therapeutic benefit for many diseases including myocardial ischemia-reperfusion injury ( Eckle *et al*., 2012; Hill *et al*., 2008; Rey *et al*., 2009), inflammatory bowel diseases ( Biddlestone *et al*., 2015; Cummins *et al*., 2013; Marks *et al*., 2015), anaemia-associated chronic kidney diseases ( Macdougall, 2008; Provenzano *et al*., 2016), wound healing ( Albina *et al*., 2001; Botusan *et al*., 2008) and assistance of organ transplantation ( Cheng *et al*., 2010). Over the years, PHD inhibitors have entered clinical trials, with FG-4592 in clinical trial phase III for the treatment of anaemia associated with chronic kidney diseases ( Provenzano *et al*., 2016). Recent studies have identified PHD inhibitors or the knockout of *VHL* as protective during mitochondrial dysfunction ( Jain *et al*., 2016). VHL inhibitor VH298 has been demonstrated for the first time *in vivo* to accelerate healing and maturation of enthuses in rats ( Qiu *et al*., 2018), highlighting a therapeutic potential of the inhibitor in wound healing.

Considering the pharmacological use and therapeutic potential of PHD inhibitors and the newly emerging VHL inhibitors, it is important to identify gene expression responses elicited by such agents. As such, we employed RNA-sequencing (RNA-seq) to determine the similarities and differences of the transcriptional response under hypoxia, the inhibitor of PHD, IOX2 ( Chowdhury *et al*., 2013), as well as the VHL inhibitor VH032 ( Frost *et al*., 2016; Galdeano *et al*., 2014). We show that IOX2 and VH032 mimic the hypoxia response and that these predominantly induce a HIF-dependent gene signature. On the other hand, hypoxia produces the broader transcriptional response amongst all the inducers used, with significant numbers of genes being induced and repressed.

## Methods

### Cell culture

Human cervix carcinoma cells HeLa (ATCC ^®^ CCL-2 ^™^) and human foreskin fibroblasts HFF (ATCC ^®^ SCRC-1041 ^™^) were obtained from the American Type Culture Collection (ATCC). All cells were propagated in Dulbecco’s Modified Eagle Medium (Sigma; 1992394) supplemented with 10% fetal bovine serum (Gibco; 10082147), 2 mM L-glutamine (Gibco; 25030024), 50 units/mL of penicillin (Lonza) and 50 µg/mL streptomycin (Lonza; DE17-602E) at 37°C. Cells were routinely tested for mycoplasma contamination using MycoAlert kit from Lonza (LT07-218).

### Treatments

For hypoxia induction, cells were incubated at 1% O _2_ in an InVIVO 300 hypoxia workstation (Ruskin Technologies). To prevent reoxygenation, cells were lysed for protein or RNA extraction in the hypoxia workstation. DMSO was used as vehicle control for compound treatment. PHD inhibitors IOX2 and FG-4592 were purchased from from Sigma (SML0652) or Selleckchem (S2919) and Selleckchem (S1007), respectively. Drugs were added to cells for the indicated length of time. VHL inhibitors VH032 and VH298 were synthesised by Pedro Soares (Ciulli lab, University of Dundee) as previously described: VH032 (ligand 7 in Galdeano *et al*. (2014); compound 1 in ( Soares *et al*., 2018) and VH298 ( Frost *et al*., 2016). VH298 was also purchased from Sigma (SML1896).

### RNA preparation for RNA-seq

HeLa cells were seeded in 35 mm dishes one day prior to treatments with 0.05% DMSO, hypoxia (1% O _2_), 250 μM IOX2, or 250 μM VH032 for 16 h. RNA was extracted using the RNeasy Mini Kit (Qiagen; 74104) according to manufacturer’s instruction. Genomic DNA was removed from RNA samples using RNase-free DNase from Qiagen (79254) as per manufacturer’s protocol. Experiments were performed in triplicates.

### RNA-seq library preparation and sequencing

Library preparation and sequencing were performed by the University of Dundee Genome Sequencing Unit. The library was prepared using TruSeq Stranded Total RNA Library Preparation Kit with Ribo-Zero ^TM^ Human/Mouse/Rat kit (Illumina; RS-122 2201) to remove ribosomal RNA (rRNA). RNA ERCC ExFold RNA Spike-In Mix (Mix1 and Mix2) was distributed throughout the RNA-seq experiment according to manufacturer’s protocol (4456739, Thermo Scientific). Paired-end Illumina sequencing was performed on the NextSeq 500 platform.

### RNA-seq data analysis

The raw sequence reads from each replicate were aligned to the Ensembl human genome GRCh37 and ERCC sequence with STAR version 2.4.2a. The aligned reads were combined and number of reads for each gene was counted with subread-featureCounts pipeline version 1.4.6-p4 ( Liao *et al*., 2014). The files were found to contain ribosomal DNA (rDNA) contaminations, the majority of which were the following two mitochondrial DNA: ENSG00000211459 and ENSG00000210082 – which were removed manually. Differential gene expression analysis was performed by the R package edgeR (v3.24.1) according to its user guide ( Robinson *et al*., 2010), and differentially expressed genes were identified at FDR of <0.05 and log2 fold change > 0.58.

Integrative analysis was performed manually in R (v1.1.453) to obtain lists of genes that overlap to publicly available datasets. Briefly, a list of differentially expressed genes upregulated in hypoxia, IOX2, VH032 or in all three conditions was compared to publicly available data and overlapping genes were exported into excel sheet. Enrichment analysis of transcription factors and chromatin binding proteins on our datasets was carried out using TFEA.ChIP (v1.2.2) according to its user guide ( Puente-Santamaria & Del Peso, 2019). Gene set enrichment analysis was performed using GSEA MSigDB online tool ( Liberzon *et al*., 2011; Subramanian *et al*., 2005) for hallmark genes with FDR < 0.05 and p value < 0.05.

Sequence data are available from Gene Expression Omnibus GSE120675.

### Quantitative real time-PCR (qRT PCR)

Total RNA extracted using the RNeasy Mini Kit (Qiagen) was reverse transcribed using the iScript™ cDNA Synthesis Kit (BIO RAD; 170-8891). SYBR green-based qRT-PCR was performed in 96-well plate using iQ™ SYBR® green supermix (BIO-RAD; 1708880) in MX3005P qPCR platform (Stratagene/Agilent). Relative quantity or fold change comparing each treatment to DMSO control for the same gene within the replicate (with the exception for *CA9* in which fold changes were calculated comparing to hypoxia) were generated using the MxPro qPCR software (v4.10), based on the ΔΔCT method according to its manual. mRNA level of β-Actin was used for normalisation. Results were shown as mean and SEM of a minimum of three independent experiments. Primers were designed and purchased from Invitrogen. Sequences of primers used are as follows: β-Actin_F, CCCAGAGCAAGAGAGG and β-Actin_R, GTCCAGACGCAGGATG; BNIP3_F, GCCCACCTCGCTCGCAGACAC and BNIP3_R, CAATCCGATGGCCAGCAAATGAGA; BNIP3L_F, GTGGAAATGCACACCAGCAG and BNIP3L_R, CTTGGGTGGAATGTTTTCGG; CA9_F, CTTTGCCAGAGTTGACAGG and CA9_R CAGCAACTGCTCATAGGCAC; FAM117B_F, CTCTTGCTGCACCGTATCTT and FAM117B_R, CATGCACTCTCTGTCTGTGTAG;GLUT3_F, CAATGCTCCTGAGAAGATCAAA and GLUT3_R, AAAGCGGTTGACGAAGAGT; HK2_F, AGCCCTTTCTCCATCTCCTT and HK2_R, AACCATGACCAAGTGCAGAA; IDH2_F, AGACCGACTTCGACAAGAATAAG and IDH2_R, GACTGCACATCTCCGTCATAG; JMJD1A_F, GTCAACTGTGAGGAGATTCCAGC and JMJD1A_R, AACTTCAACATGAATCAGTGACGG; JMJD2B_F, GGGGAGGAAGATGTGAGTGA and JMJD2B_R, GACGGCTTTTGGAGGGTAAT; JMJD2C_F, CGAGGTGGAAAGTCCTCTGAA and JMJD2C_R GGGCTCCTTTAGACTCCATGTAT; JMJD6_F, TGGCATGTTGTCCTCAATCT and JMJD6_R, TCTCCCTCTTACCGTCTTGT; NDRG1_F, GGAGTCCTTCAACAGTTTGG and NDRG1_R, CACCATCTCAGGGTTGTTTAG; PHD2_F, GAAAGCCATGGTTGC and PHD2_R, TGTCCTTCTGGAAAAATTCG; PHD3_F, ATCGACAGGCTGGTCCTCTA and PHD3_R, CTTGGCATCCCAATTCTTGT; RNF187_F, GGGTCTGTGGAAATCATGAGAA and RNF187_R, CAGCTTCTTGTAGTCGGTCAG

### Immunoblotting

Cells were harvested using radio Immunoprecipitation assay (RIPA) lysis buffer (50 mM Tris pH 8, 150 mM NaCl, 0.1% (w/v) SDS, 1% (v/v) NP-40, 0.5% (w/v) sodium deoxycholate, 5 mM NaF, 500 mM Na _3_VO _4_, and one tablet/10 mL Complete, mini, EDTA-free protease inhibitor [Roche; 11873580001]) and kept on ice for 15–30 min before centrifugation at 17,000 × g, 4°C for using Heraeus™ Fresco™ 21 Microcentrifuge (Thermo Scientific) 10 min. The supernatant was collected and stored at –80°C. SDS PAGE and immunoblots were carried out using standard protocols ( Frost *et al*., 2016).

The following primary antibodies were used for immunoblotting (catalogue number, supplier, clonality, host species and dilution factor were included): HIF-1α (610958, BD Biosciences; monoclonal; mouse; 1:1000), β-Actin (66009-1-Ig, Proteintech; monoclonal; mouse; 1:10000), BNIP3 (ab10433, Abcam; monoclonal; mouse; 1:10000), BNIP3L (12396, Cell Signalling; monoclonal; rabbit; 1:1000), CA9 (NB100-417, Novus Biologicals; polyclonal; rabbit; 1:1000), GLUT1 (12939, Cell Signalling; monoclonal; rabbit; 1:1000), GLUT3 (LS-C176045, LSBio; polyclonal; mouse; 1:1000), HK2 (2867S, Cell Signalling; monoclonal; rabbit; 1:2000), JMJD1A (ABE195, Millipore; polyclonal; rabbit; 1:1000), JMJD2B (8639S, Cell Signalling; monoclonal; rabbit; 1:1000), JMJD2C (PA5-23065, Thermo Scientific; polyclonal; rabbit; 1:1000), NDRG1 (5196, Cell Signalling; polyclonal; rabbit; 1:1000), PHD2 (A300-322A, Bethyl Laboratories; polyclonal; rabbit; 1:1000).

## Results

### IOX2 and VH032 induce a similar transcriptional response profile, while hypoxia induces a broader response in cells.

The hypoxia inducible factors (HIFs) can be induced in a variety of different ways, from the physiological inducer of low oxygen, to the pharmacological inhibition of proteins involved in the HIF pathway, as well as by changes in alternative pathways such as transcription and translation ( Ferreira *et al*., 2015; Moniz *et al*., 2015; Semenza, 2003).

To understand the similarities and differences between the transcriptional responses to several HIF inducers, an unbiased high-throughput RNA-sequencing (RNA-seq) was performed. Human cervical cancer HeLa cells were exposed to 0.05% DMSO (vehicle control), hypoxia (1% O _2_), PHD inhibitor IOX2 or VHL inhibitor VH032 for 16 hours prior to profiling for global transcriptomic analysis using RNA seq.

**Figure 1.**
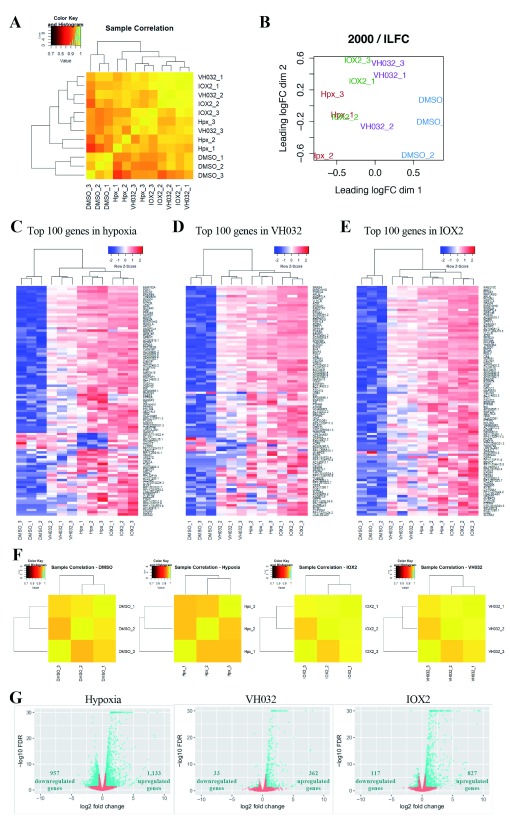
Differential gene expression analysis of RNA-seq results. ( **A**) Heatmap of Pearson correlations among RNA-seq samples that have been normalised to their total counts. ( **B**) Multidimensional scaling plot of RNA-seq data. The distance between two samples reflects the leading logFC of the corresponding samples. The leading logFC is the average (root mean square) of the 2000 largest absolute logFCs for genes between those two samples. ( **C–E**) Heatmaps of log2 counts per million (logcpm) across all the samples using the top 100 most differentially expressed (DE) genes in ( **C**) Hypoxia, ( **D**) VH032, and ( **E**) IOX2. The Pearson correlation was used to compute distances between genes and samples, and the clustering was performed using average linkage. Each column corresponds to a sample and each row corresponds to a specific gene. ( **F**) Heatmaps of Pearson correlations between replicates of the same conditions. Each data had been normalised to their total counts. ( **G**) Each dot represents a differentially expressed gene comparing the condition stated in the heading legend to DMSO vehicle control. Blue dots represent genes with increased expression (logFC > 0.58; to the right) or decreased expression (logFC < –0.58; to the left) at false discovery rate (FDR)<0.05. Blue triangles (present at –log10 FDR of 30) represent genes with logFC > 0.58 or < –0.58 and –log10 FDR > 30.

Differential expression analysis of data collected from RNA-seq showed that DMSO samples cluster together with weak correlation to the other treatments, whilst hypoxia, IOX2 and VH032 conditions were grouped close to one another ( Figure 1A–B). Furthermore, heatmaps generated using the top 100 most differentially expressed (DE) genes in each experimental condition comparing to DMSO control showed that hypoxia ( Figure 1C), VH032 ( Figure 1D) and IOX2 ( Figure 1E) displayed similar transcriptional profiles to each other and were noticeably distinct from the vehicle control DMSO. These observations are likely due to the activation of HIF pathway as the three treatments activate HIF. Correlation analysis heatmaps for each condition showed a strong correlation between 0.95 and 1 across replicates of the same experimental condition ( Figure 1F), and replicates of each treatment cluster together in heatmaps of top 100 most DE genes ( Figure 1C–E), demonstrating that replicates of each condition were similar and statistically close to each other.

To investigate and observe the differences in gene expression between treatments, volcano plots were generated ( Figure 1G). Overall, hypoxia exposure induced the broadest transcriptional changes, followed by IOX2 and finally VH032 resulted in the narrowest profile ( Figure 1G). Analysis revealed the presence of more than 2,000 genes that were differentially expressed at 5% false discovery rate (FDR) in hypoxia, with similar numbers of genes being upregulated (1,133; Dataset 1 ( Frost, 2019)) and downregulated (957; Dataset 1 ( Frost, 2019) ( Figure 1G)). Treatments of cells with IOX2 or VH032 induced mostly upregulation of genes (827 in IOX2 and 362 in VH032, Dataset 1 ( Frost, 2019)), and only 117 (IOX2; Dataset 1 ( Frost, 2019)) and 33 (VH032; Dataset 1 ( Frost, 2019)) genes were found to be repressed ( Figure 1G).

To investigate the nature of the transcriptional data we obtained, we performed integrative analysis of our hypoxia dataset with publicly available hypoxia-inducible gene sets ( Table 1, Dataset 1 ( Frost, 2019)). Overlap analysis showed that 36% (410 out the 1,133) genes upregulated in hypoxia were present in at least one of the reported datasets, with 115 genes found upregulated in 16 cell lines ( Ortiz-Barahona *et al*., 2010), 129 genes in HeLa dataset ( Mense *et al*., 2006), as well as 75 and 307 genes in in MCF7 cells ( Chan *et al*., 2016; Elvidge *et al*., 2006). This analysis confirmed the cell-specific and time-dependent transcriptional responses elicited by hypoxia exposure in cells. We also compared our IOX2 and VH032 datasets with the previously reported gene sets to assess the extent to which genes upregulated in IOX2 or VH032 also hypoxia-inducible. We identified a large portion of genes upregulated by IOX2 (39%; 325 out of 827) or VH032 (56%; 200 out of 362) to be present in at least one of these reported hypoxia datasets ( Table 1, Dataset 1 ( Frost, 2019)). This analysis showed that VH032 is predominantly regulating hypoxia-inducible genes, consistent with specific on-target effects on VHL ( Frost *et al*., 2016).

**Table 1.** Comparison to reported hypoxia-inducible datasets.

Description of dataset	Number of genes in the dataset	Number of upregulated genes				Reference
		Hypoxia (1133)	IOX2 (827)	VH032 (362)	Overlap (306)	
Hypoxia-inducible in 16 cell lines	259	115	92	69	64	( Ortiz-Barahona *et al*., 2010); Supporting data G
HeLa hypoxia- inducible	1141	129	100	61	55	( Mense *et al*., 2006); Supporting data H
MCF7 hypoxia- inducible_Elvidge2006	246	75	53	45	42	( Elvidge *et al*., 2006); Supporting data I
MCF7 hypoxia-inducible_Chan2016	1081	307	257	172	163	( Chan *et al*., 2016); Supporting data J
**TOTAL**		**410**	**326**	**200**	**185**	

### Genes upregulated in hypoxia, IOX2 and VH032 are HIF-dependent

**Figure 2.**
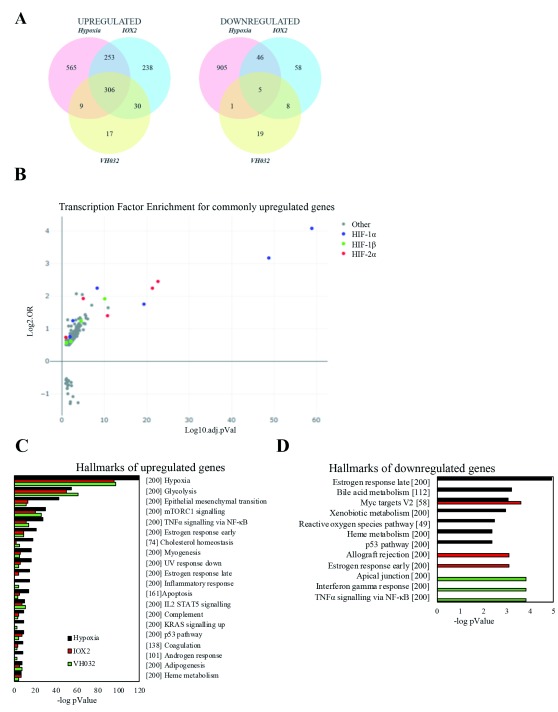
Analysis of differential expressed genes obtained by RNA-seq. ( **A**) Venn diagrams showing the number of genes upregulated (logFC > 0.58) or downregulated (logFC < –0.58) with false discovery rate (FDR) < 0.05 in hypoxia, IOX2 and VH032 treated cells compared to DMSO control. ( **B**) Transcription factor enrichment analysis using TFEA.ChIP showing binding site enrichment for genes commonly upregulated in hypoxia, IOX2 and VH032. The graph represents the adjusted p value (-log10 FDR) and the log-odds ratio (Log2.OR) for the association of ChIP datasets. ( **C–D**) Gene set enrichment analysis (GSEA) MsigDB showing significant enrichment of gene set signatures for ( **C**) downregulated and upregulated genes in hypoxia, IOX2 or VH032 and ( **D**) genes upregulated in hypoxia, IOX2 and VH032 at 5% FDR.

Comparative analysis of upregulated genes distinctly showed that the majority of VH032-induced genes (~87%; 315 out of 362) are also upregulated by hypoxia ( Figure 2A). On the other hand, IOX2-induced genes are only partially shared with the hypoxia signature (~68%; 559 out of 827). Notably, nearly all of the VH032-induced genes (93%) are shared with IOX2. Overall, 306 genes are upregulated in all of the three experimental conditions ( Figure 2A; Dataset 1 ( Frost, 2019)).

Given that hypoxia, IOX2, and VH032 all induce HIF transcriptional activity, we next investigated the extent to which these 306 overlapped genes upregulated in all three conditions were regulated by HIF transcription factors. We performed integrative analysis on the overlapped genes with reported datasets of validated HIF-1 targets ( Benita *et al*., 2009), as well as HIF-1α and HIF-2α binding sites under hypoxia in MCF7 ( Mole *et al*., 2009) and HepG2 ( Smythies *et al*., 2019) identified in ChIP-sequencing experiments ( Table 2, Dataset 1 ( Frost, 2019)). This analysis revealed that 132 out of these 306 (43%) genes were HIF-dependent ( Table 2, Dataset 1 ( Frost, 2019)). A total of 33 out of the 306 shared genes was present in the list of 93 validated HIF-1 target genes (Dataset 1 ( Frost, 2019)). Analysis using MCF7 ChIP-seq dataset showed that 62 (20%) and 33 (11%) of the 306 upregulated genes contained HIF-1α and HIF-2α binding sites, respectively (Dataset 1 ( Frost, 2019)). A higher level of overlap was observed when we analysed the HepG2 ChIP-seq dataset, revealing that 90 out of the 306 genes (29%) contained either HIF-1α or HIF-2α binding sites (Dataset 1 ( Frost, 2019)).

**Table 2.** Hypoxia-inducible factors (HIF) dependency analysis. Our datasets were compared to reported list of validated HIF target genes and ChIP-seq datasets of HIF-1α and HIF-2α binding sites.

Description of dataset	Number of genes in the dataset	Number of upregulated genes				Reference
		Hypoxia (1133)	IOX2 (827)	VH032 (362)	Overlap (306)	
HIF-1 target	93	49	39	36	33	( Benita *et al*., 2009); Supporting data L
HIF-1α binding sites (MCF7)	356	101	86	65	62	( Mole *et al*., 2009); Supporting data M
HIF-2α binding sites (MCF7)	301	65	40	35	33	( Mole *et al*., 2009); Supporting data N
HIF-1α binding sites (HepG2)	1516	153	137	94	90	( Smythies *et al*., 2019); Supporting data O
HIF-2α binding sites (HepG2)	1528	173	153	95	90	( Smythies *et al*., 2019); Supporting data P
**TOTAL**		**274**	**215**	**141**	**132**	

We next utilised TFEA.ChIP that exploits publicly available ChIP-seq datasets to perform enrichment analysis of transcription factors and chromatin binding proteins on our dataset of commonly upregulated genes ( Puente-Santamaria & Del Peso, 2019). Result demonstrated HIF-dependency of the 306 commonly induced genes as HIF transcription factors were significantly enriched. ( Figure 2B).

Taken together, these comparative analyses demonstrate the level of HIF dependency for genes upregulated by both hypoxia, IOX2 and VH032.

To investigate the cellular processes induced by hypoxia, IOX2 or VH032, gene set enrichment analysis (GSEA) was performed according to the molecular signature database (MSigDB) ( Subramanian *et al*., 2005; Liberzon *et al*., 2011). All treatments induced a similar set of enrichment for genes involved in the “cellular response to hypoxia”, “glycolysis”, “epithelial-mesenchymal transition”, “mTORC1 signalling” and “NF-κB signalling” ( Figure 2C). However, genes repressed by the different treatments mapped to quite diverse cellular pathways and responses ( Figure 2D). Furthermore, the group of 306 commonly upregulated genes in all three conditions was enriched with genes found in these same hallmarks ( Figure 2B), primarily hypoxia and glycolysis. Altogether, data indicates that the three treatments activate mainly the hypoxia signalling pathway via HIF transcription factors.

### RNA-seq validation, genes commonly upregulated in hypoxia, IOX2 and VH032

**Figure 3.**
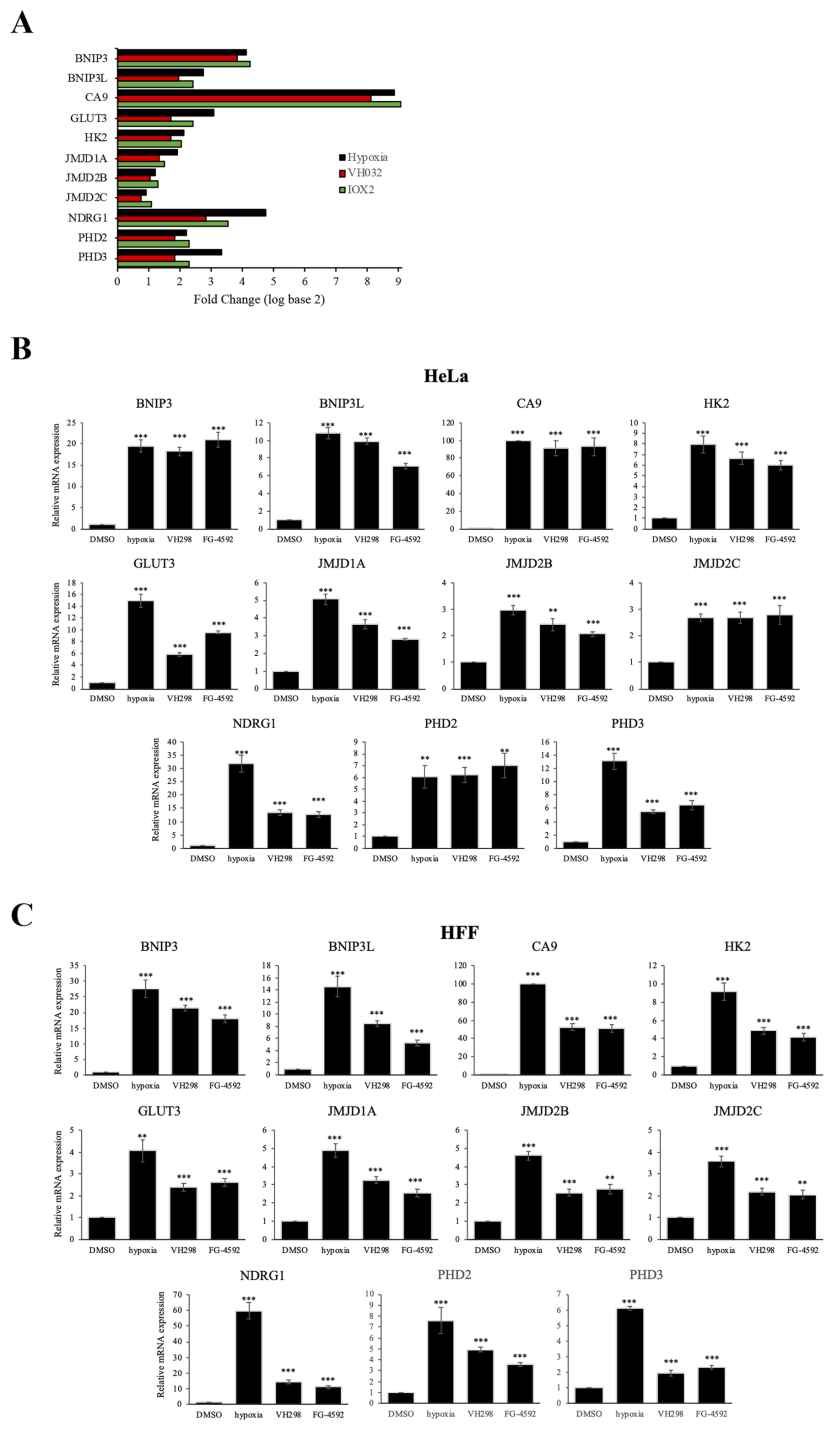
Validation of genes with increased transcript level in hypoxia, IOX2 and VH032. ( **A**) Bar plot showing log2FC according to data obtained from RNA seq analysis of known HIF target genes in hypoxia, IOX2 and VH032. ( **B**) HeLa and ( **C**) HFF cells were treated with 0.05% DMSO (vehicle control), 1% O _2_ (hypoxia), 100 µM VH298 and 50 µM FG-4592 for 16 h prior to mRNA extraction. The graphs show relative mRNA transcripts normalised to actin mRNA levels. The mean + SEM were determined from three independent experiments. Two-tailed student t-test analysis was performed * P ≤ 0.05, ** P ≤ 0.01, *** P ≤ 0.001 and ns: P>0.05.

**Figure 4.**
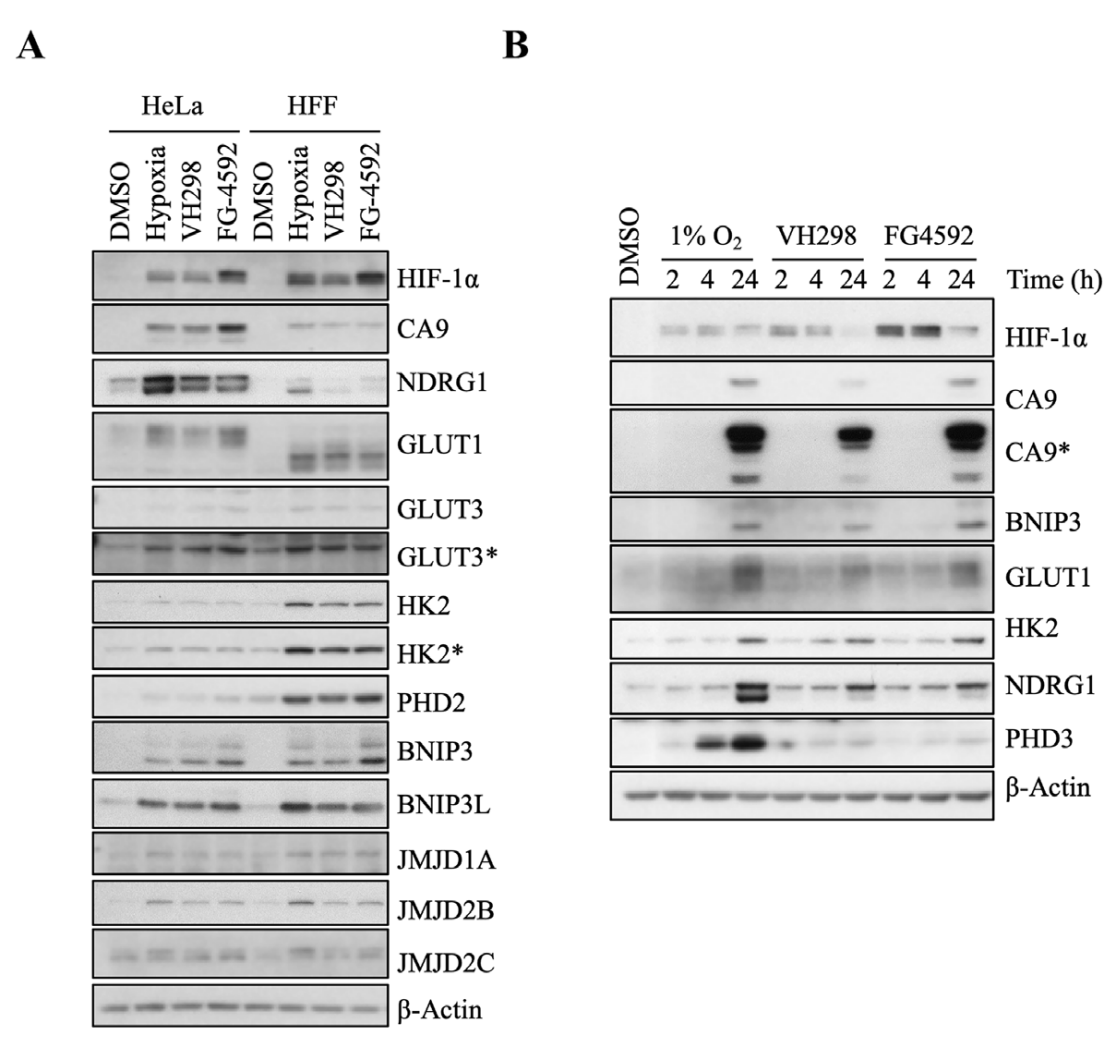
Analysis of protein levels of genes with increased transcription in hypoxia, IOX2 and VH032. HIF targets were increased in hypoxia, VH298 and FG-4592. 0.05% DMSO (vehicle control), 1% O _2_ (hypoxia), 100 µM VH298 and 50 µM FG-4592 were introduced to ( **A**) HeLa or HFF for 24 hours and ( **B**) HeLa for indicated time. Protein levels were analysed by immunoblotting using antibodies against indicated proteins, with β-Actin as loading control. The blots shown are representative of three independent experiments. * indicates longer exposure.

To validate data obtained from the RNA-seq analysis, we selected several known HIF target genes amongst the 306 upregulated genes ( *BNIP3*, *BNIP3L*, *CA9*, *GLUT3* [ *SLC2A3*], *HK2*, *JMJD1A* [ *KDM3A*], *JMJD2B* [ *KDM4B*], *JMJD2C* [ *KDM4C*], *NDRG1*, *PHD2* [ *EGLN1*], and *PHD3* [ *EGLN3*]) to perform quantitative real-time PCR (qRT PCR) ( Figure 3A). We replaced IOX2 with the PHD inhibitor FG-4592 that is currently in clinical trial phase III ( Provenzano *et al*., 2016). Furthermore, VH032 was replaced with the more potent VHL inhibitor VH298 ( Frost *et al*., 2016). After exposure to 16 hours of hypoxia, FG-4592 (50 µM) or VH298 (100 µM), mRNA levels of these genes increased significantly with similar induction profiles in both HeLa ( Figure 3B) and HFF cells ( Figure 3C). Hypoxia showed the strongest induction profiles in nearly all genes examined in both cell lines ( Figure 3). Moreover, the changes in transcript levels were also reflected at the protein level ( Figure 4). Accumulation of the products of these HIF target genes, as well as GLUT1 protein, another HIF target which we had previously characterised at mRNA level ( Frost *et al*., 2016), was detected following 24 hour treatment of hypoxia, VH298 or FG-4592 in HeLa and HFF cells ( Figure 4A). In both cell lines, the three treatments induced similar levels of protein expression for the majority of HIF targets assessed, with NDRG1 being the most prominent in hypoxia. FG-4592 was the strongest inducer of CA9 in HeLa cells and BNIP3 in both cell lines ( Figure 4A). The difference in the levels these genes and therefore proteins were increased could be due to the single time point used; particularly since the three conditions act on the HIF pathway at different stages (1% oxygen level limits the activities of PHD and FIH, FG 4592 inhibits PHDs and VH298 inhibits VHL downstream of hydroxylation by PHD). To address this question, we performed a time course analysis for the three inducers and investigated protein levels of the different HIF-target genes ( Figure 4B). This revealed that hypoxia is the strongest inducer of all the proteins we have analysed at the 24 h post-treatment time point.

### RNA-seq validation, genes solely upregulated in hypoxia and IOX2.

**Figure 5.**
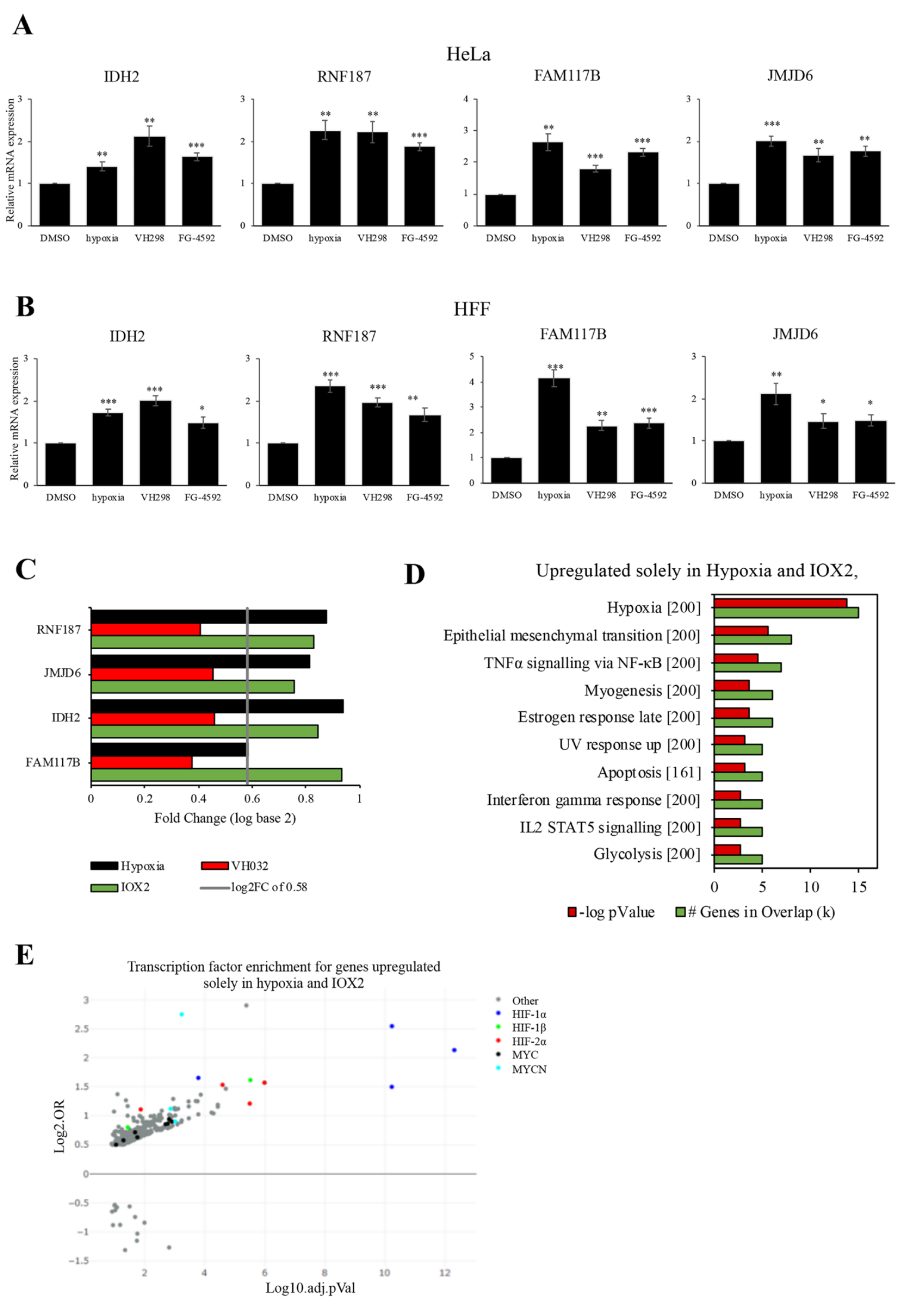
RNA seq validation of genes solely upregulated in hypoxia and IOX2, but not VH032. ( **A**) HeLa and ( **B**) HFF cells were treated with 0.05% DMSO (vehicle control), 1% O _2_ (hypoxia), 100 µM VH298 and 50 µM FG-4592 for 16 h prior to mRNA extraction. The graphs show relative mRNA transcripts normalised to actin mRNA levels. The mean + SEM were determined from three independent experiments. Two-tailed student t-test analysis was performed * P ≤ 0.05, ** P ≤ 0.01, *** P ≤ 0.001 and ns: P>0.05. ( **C**) Table showing log2FC according to data obtained from RNA-seq analysis of known HIF target genes in hypoxia and IOX2, but not VH032. ( **D**) Gene set enrichment analysis (GSEA) MsigDB showing significant enrichment of gene set signatures for genes upregulated in hypoxia and IOX2, but not found in VH032 at 5% false discovery rate (FDR). ( **E**) Transcription factor enrichment analysis using TFEA.ChIP showing binding site enrichment for genes upregulated in hypoxia and IOX2, but not B032. The graph represents the adjusted p value (-log10 FDR) and the log-odds ratio (Log2.OR) for the association of ChIP datasets.

Hypoxia and IOX2 share the larger overlap of 252 upregulated genes that are not found in VH032 (Dataset 1 ( Frost, 2019)). On the other hand, there are 30 upregulated common genes between IOX2 and VH032, but not hypoxia, as well as the 9 common upregulated genes in hypoxia and VH032, but not IOX2 ( Figure 2A). As recent studies have revealed additional targets of PHD enzymes, we analysed several of these 252 genes to determine whether PHDs induce transcriptomic changes independent of HIF activity. We selected four genes, including *IDH2*, *RNF187*, *FAM117B* and *JMJD6* from the list of 252 genes upregulated solely in hypoxia and IOX2 for validation by qRT-PCR. The results, however, show that mRNA levels of these genes increased significantly in all the three conditions, including the VHL inhibitor VH298 ( Figure 5A–B). Analysis of the RNA-seq data revealed an increase in each of the four genes in VH032 treatment (Dataset 1 ( Frost, 2019)); however, this level was insufficient to reach the threshold of log2FC of 0.58 ( Figure 5C). As VH298 is more potent than VH032 ( Frost *et al*., 2016), VH298 is predicted to induce a more pronounced effect on gene expression of target genes. It is likely that the 252 upregulated genes were found to be enriched solely in hypoxia and IOX2, are also induced by the more potent VHL inhibitor VH298, indicative of a common regulator between these treatments. Furthermore, these 252 genes showed significant enrichment of genes involved in pathways similar to commonly upregulated genes ( Figure 5D, Figure 2B), as well as enriched for HIF binding sites ( Figure 5E).

## Discussion

Here, we used high-throughput RNA-sequencing to investigate the similarity and differences in the transcriptional response towards hypoxia, the PHD inhibitor IOX2 and the VHL inhibitor VH032. Although genome-wide expression profiling comparing hypoxia and IOX2 has previously been reported ( Chan *et al*., 2016), to our knowledge this is the first report of gene expression profiling comparing side-by-side responses of hypoxia and PHD inhibitors to VHL inhibitors. These three treatments activate the HIF transcription factors, but via limiting or inhibiting different components of the hypoxia signalling pathway.

Our results provide insights into the effects of inhibiting PHD or VHL on HIF target genes, and unique responses in each condition. While hypoxia induced the broadest transcriptional changes, IOX2 and VH032 possessed similar transcriptional responses. The three conditions upregulated a common group of 306 genes (Dataset 1 ( Frost, 2019)), the majority of which are regulated by HIF transcription factors ( Figure 2B). From this list, we were able to validate a number of known HIF targets in HeLa and HFF cells ( Figure 3, Figure 4). Furthermore, we also found that 132 of these 306 genes were either validated HIF targets or possess HIF-1α/2α binding sites (Dataset 1 ( Frost, 2019)). This suggest that while the 132 genes are likely HIF targets, the remaining 174 genes (Dataset 1 ( Frost, 2019)) could also be potential novel HIF targets.

**Figure 6.**
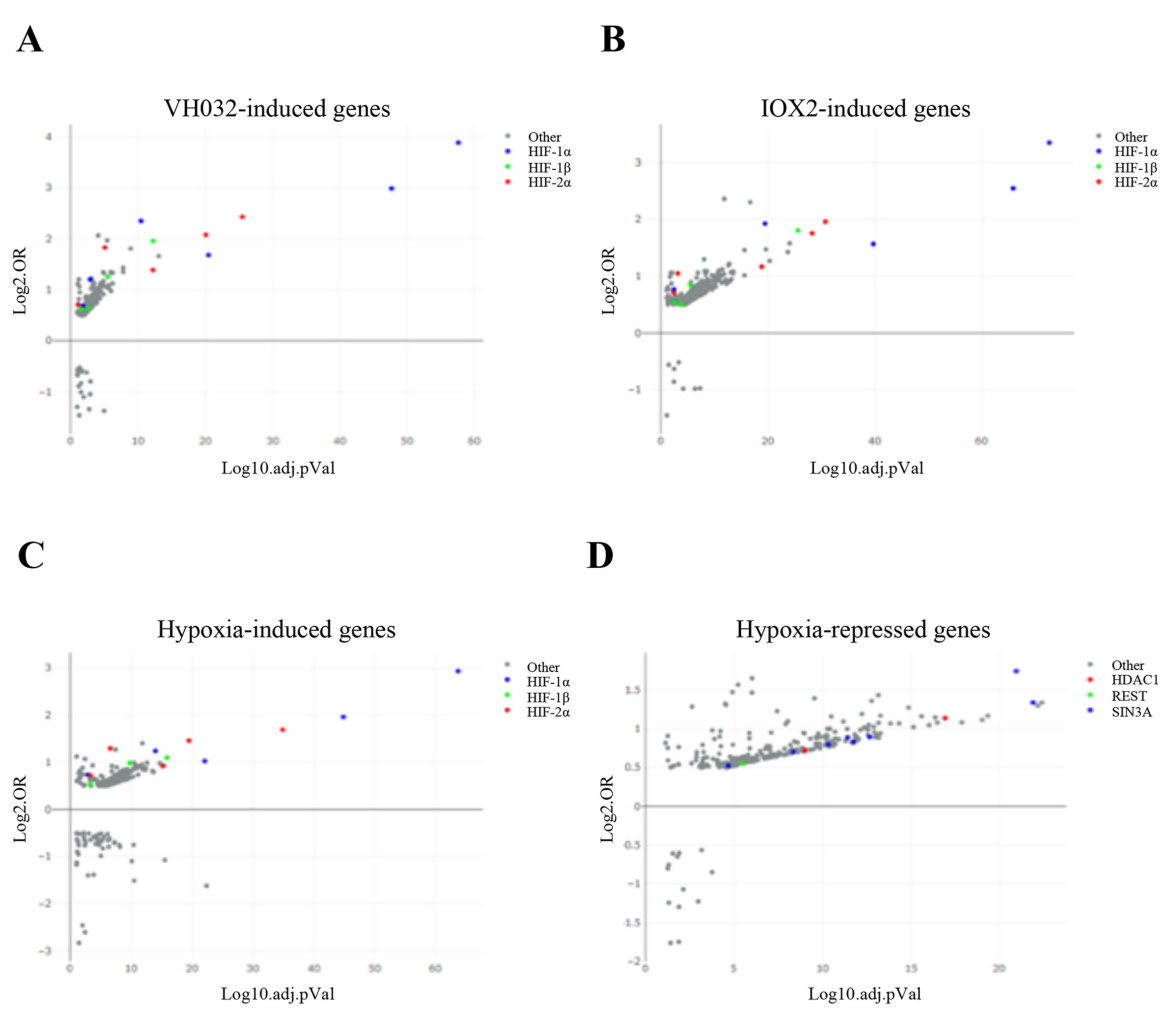
Transcription factor enrichment analysis. Transcription factor enrichment analysis using TFEA.ChIP showing binding site enrichment for genes upregulated in ( **A**) VH032, ( **B**) IOX2 and ( **C**) hypoxia, or ( **D**) downregulated in hypoxia. The graph represents the adjusted p-value (-log10 false discovery rate (FDR)) and the log-odds ratio (Log2.OR) for the association of ChIP datasets.

As hypoxia, VH032 and IOX2 activate HIF, our datasets of genes induced in these conditions are predominantly enriched for HIF transcription factors ( Figure 6A–C). Beside gene activation, hypoxia also promotes gene repression. Our results show that hypoxia downregulated a significantly larger number of genes compared to IOX2 and VH032 ( Figure 1G). Various mechanisms of transcriptional repression in hypoxia are known ( Batie *et al*., 2018) and one mechanism includes the activity of SIN3A. A recent bioinformatics study showed that SIN3A and a number of its co-repressors including HDAC1 were overrepresented in the proximity of genes transcriptionally repressed by hypoxia ( Tiana *et al*., 2018). Consistent to the reported roles of SIN3A and HDAC1 in hypoxia signalling ( Batie *et al*., 2018), we found that our datasets of downregulated genes in response to hypoxia were enriched for SIN3A and HDAC1 ( Figure 6D). The transcription factor REST was also enriched in genes repressed in hypoxia ( Figure 6D) and this is consistent to a recent finding that REST transcriptionally repressed genes in hypoxia ( Cavadas *et al*., 2016).

Geneset enrichment analysis suggests that hypoxia, VH032 and IOX2 commonly upregulated genes enriched for NF-κB signalling ( Figure 2B). NF-κB is a transcription factor that has been shown to respond to cellular stress, including hypoxia and PHD inhibition ( Cummins *et al*., 2006). Under hypoxia, NF-κB is activated and induces increased angiogenesis and decreased apoptosis ( D'Ignazio & Rocha, 2016).

Overall, we reveal for the first time a comparison of genome-wide gene expression analysis of HIF activators, including the physiological inducer hypoxia, and small molecule inhibitors of PHD enzymes and VHL. Understanding the differential regulation of genes in response to the three conditions will help to determine the functions of PHD and VHL in hypoxia signalling, as well as revealing novel HIF-dependent and –independent genes. Furthermore, considering the potential use of PHD inhibitors that are currently in clinical trials and the potential of VHL inhibitors for therapeutic benefits, our report contributes to the further understanding of the pharmacological effects of these inhibitors.

## Data availability

### Underlying data

Underlying data for this study is available from Open Science Framework (OSF)

OSF: Dataset 1. RNA-seq analysis of PHD and VHL inhibitors reveals differences and similarities to the hypoxia response https://doi.org/10.17605/OSF.IO/4A6TG ( Frost, 2019)

Licence: CC0 1.0 Universal

Legend for file Supporting data.xlsx

A – List of genes upregulated in hypoxia with FDR ≤ 5% and logFC ≥ 0.58

B – List of genes downregulated in hypoxia with FDR ≤ 5% and logFC ≤ –0.58

C – List of genes upregulated in IOX2 with FDR ≤ 5% and logFC ≥ 0.58

D – List of genes upregulated in VH032 with FDR ≤ 5% and logFC ≥ 0.58

E – List of genes downregulated in IOX2 with FDR ≤ 5% and logFC ≤ –0.58

F – List of genes downregulated in VH032 with FDR ≤ 5% and logFC ≤ –0.58

G – List of hypoxia-inducible genes conserved across 16 cell lines ( Ortiz-Barahona *et al*., 2010)

H – List of hypoxia-inducible genes in HeLa ( Mense *et al*., 2006)

I – List of hypoxia-inducible genes in MCF7 ( Elvidge *et al*., 2006)

J – List of hypoxia-inducible genes in MCF7 ( Chan *et al*., 2016)

K – List of genes commonly upregulated in hypoxia, IOX2, and VH032. Genes that are found in HIF 1 validated target genes (K), HIF 1α (L) and HIF 2α (M) binding sites in MCF7, as well as HIF 1α (N) and HIF 2α (O) in HepG2 are highlighted in yellow under column C-G, respectively

L – HIF 1 validated target genes ( Benita *et al*., 2009)

M – List of HIF-1α binding sites identified in MCF7 ( Mole *et al*., 2009)

N – List of HIF-2α binding sites identified in MCF7 ( Mole *et al*., 2009)

O – List of HIF-1α binding sites identified in HepG2 ( Smythies *et al*., 2019)

P – List of HIF-2α binding sites identified in HepG2 ( Smythies *et al*., 2019)

Q – List of genes solely upregulated in hypoxia and IOX2, but not in VH032. However, LogFC value in VH032 is stated in column C, together with its p-Value (column D) and FDR (column E)

Sequence data from this study has been uploaded to Gene Expression Omnibus, accession number: GSE120675
