# Supplementary material for: RNA-seq analysis of PHD and VHL inhibitors reveals differences and similarities to the hypoxia response
Source: Wellcome Open Res. 2019 Jan 29;4:17. [Version 1] doi: 10.12688/wellcomeopenres.15044.1 (PMC6376255; doi:10.12688/wellcomeopenres.15044.1)
Supplement: Peer review history

# Peer review: Referee response for version 1

**DOI:** 10.21956/wellcomeopenres.16413.r34715

**Published:** 2019-02-13

**Authors:** Cormac T. Taylor

**Affiliations:** 1 UCD Charles Institute of Dermatology, School of Medicine and Medical Science, University College Dublin, Dublin, Ireland

In this manuscript, Frost *et al* have used high-throughput RNA sequencing to compare the cellular transcriptional response to hypoxia with that of pharmacologic HIF hydroxylase or pVHL inhibition. Of interest, hypoxia was a more potent stimulus of gene expression and repression than either hydroxylase or pVHL inhibition. Given the recent introduction of pharmacologic targeting of the HIF hydroxylases  into clinical medicine, understanding the cellular consequences of exposure to these agents in terms of global gene expression (while already studied to some degree) is of continuing interest. The manuscript is well written, addresses a clear and interesting hypothesis and delivers clear and important data. The experiments are controlled and appear to be well carried out. The introduction includes a nice up to date review of the pharmacology of the HIF pathway.

**Specific Comments:**

 The authors should discuss the choice of IOX2 as a pharmacologic hydroxylase inhibitor for the transcriptomic aspect of the study. What was the rationale of choosing this inhibitor over some of the more commonly used and well characterized agents such as DMOG. Furthermore, the authors should explain why the use of IOX2/VH032 was changed for FG-4592/VH298 for the RT-PCR validation studies (would it not have made more sense to stick with the same compound).

 It should be made clear why Hela cells and not a more physiologically relevant cell line were used for the transcriptomic experiments. As these studies involve pharmacologic and hypoxic treatments, could primary cells not have been used? Hela cells carry many chromosomal abnormalities and have limited relevance to normal cell function.  This limitation should be discussed.

 In Figure 1, it would be good to demonstrate (e.g. by western blot or HRE-liuciferase reporter assay or both) the distinct temporal profile of HIF-1 and HIF-2  expression/activity elicited by the three treatments in Hela cells over the 16 hour time course of exposure. It is possible that these treatments (due to their differential mechanisms) elicit quite different temporal changes in HIF expression which may be of relevance to the genes expressed.

I have read this submission. I believe that I have an appropriate level of expertise to confirm that it is of an acceptable scientific standard.

# Peer review: Referee response for version 1

**DOI:** 10.21956/wellcomeopenres.16413.r34717

**Published:** 2019-02-11

**Authors:** James A Nathan

**Affiliations:** 1 Department of Medicine, Cambridge Institute for Medical Research, University of Cambridge, Cambridge, UK

Frost et al examine how HIF transcriptional responses vary depending on the mode of HIF activation – hypoxia versus inhibition of HIF-α degradation. Using RNAseq the authors find that hypoxia, PHD inhibition or VHL inhibition activate the same HIF dependent genes but with some differences, principally relating to gene suppression observed in hypoxia. The quality of the data is high and the experiments are well presented. The results substantiate findings in previous reported studies and provide a very useful dataset of hypoxia and/or HIF responsive genes. In addition, they demonstrate a clear difference between the effect of hypoxia on gene repression compared to pharmacological stabilisation of HIF-α. Overall, these findings will be of significant interest to many researchers within the field.

 I have only one minor comment regarding the relative stabilisation of HIF-α in response to the different treatments used. The RNAseq analysis was performed on cells treated for 16 hr with 1% oxygen, 250 μM IOX2 or 250 μM VH032. Could the authors comment on the level of HIF-α stabilisation in response to these different treatments? Figure 4B suggests that HIF-1α stabilisation varies over time in a distinct manner with pharmacological inhibition compared to hypoxia. Are similar findings observed for IOX2 and VH032? It would also be helpful if the authors could comment on how HIF-2α may vary following these different treatments.

I have read this submission. I believe that I have an appropriate level of expertise to confirm that it is of an acceptable scientific standard.
